# Influence of Two Root Media and Three Vermicompost Amendments on Bacterial Communities in a Greenhouse Container Garden Model System

**DOI:** 10.3390/microorganisms13081885

**Published:** 2025-08-13

**Authors:** Sihan Bu, Nikita H. Nel, Alyssa W. Beavers, Kameron Y. Sugino, Katherine Alaimo, John A. Biernbaum, Sarah S. Comstock

**Affiliations:** 1 Department of Food Science and Human Nutrition, Michigan State University, East Lansing, MI 48824, USA; 2 Department of Nutrition and Food Science, Wayne State University, Detroit, MI 48201, USA; 3 Department of Horticulture, Michigan State University, East Lansing, MI 48824, USA

**Keywords:** peat-lite, compost, vermicompost, bacterial communities, greenhouse, rhizosphere

## Abstract

The aim of this study was to determine the impact of two root media and three vermicompost amendments on the root zone bacterial communities and harvest mass of lettuce grown in a greenhouse container garden model system. Lettuce seeds were planted in seven root media/amendment conditions. Lettuce was later harvested, and root media DNA was extracted for 16S rRNA sequencing to determine the composition of, as well as the alpha and beta diversity of, the bacterial communities. Fresh weight, dry weight, and percentage dry weight of lettuce were calculated under each treatment. Results indicate that the peat-lite growth media without any additions had the lowest rhizosphere bacterial alpha diversity compared to the other six growth media. Bacterial communities from containers with peat-lite media were significantly different than those from containers with compost-based media as measured by beta diversity. Moreover, the compost-based medium with vermicompost condition tended to result in a higher percentage dry weight lettuce than lettuce grown under the peat-lite condition. The peat-lite treatment condition had the numerically lowest dry weight (%) and bacterial diversity. Addition of vermicompost amendments had varying impacts on bacterial diversity, bacterial community composition, and harvest mass. Overall, this experiment establishes a protocol which can be applied for further understanding of the impact of root media type and vermicompost amendments on rhizosphere bacterial diversity and harvest mass.

## 1. Introduction

Rapid urbanization and the use of pesticides, as well as reduced plant biodiversity, have drastically altered the impact of soil microbes on the human microbiome [1]. Due to the separation between the outdoors and living environments, urban populations face reduced exposure to environmental microbes that exist in the soil. As new research continues to support the importance of establishing a healthy gut microbiome in childhood, scientists are seeking to understand how geographic differences play a role in early colonization [2,3]. Urban families living in cities experience a loss of contact with outdoor-associated microbes, which results in a less diverse gastrointestinal microbiome and increased incidence of auto-immune disorders [4,5]. Evidence suggests that introducing natural soil biodiversity into urban environments can support immunoregulation and decrease the abundance of pathogenic bacteria in children [6]. In an effort to enhance the health of urban populations, an increased interest has been placed in identifying specific soil microbes and amendments that could potentially contribute to positive human health effects.

To form a foundational understanding of how soil microbes interact with the environment, it is important to characterize how different soil amendments affect plants and change the soil and plant microbial communities. Soil is the source of microbes for the rhizosphere, a microorganism rich area of 1–2 mm surrounding the roots of a plant [7]. The bacterial diversity levels of the rhizosphere can influence plant growth through altering nutrient absorption or providing protection against disease [8]. Potting media or external amendments, such as compost, have been shown to impact the composition of rhizosphere microbial communities [9,10]. When added to the root zone of a plant, suppressive compost promotes the growth of beneficial bacteria that inhibit the proliferation of potential pathogens and increases the total microbial biomass [11]. Beyond the type of compost used, the interactions between the rhizosphere and its microbes are known to be impacted by soil type, cultivation age, climate, and type of plant species being grown [12,13,14,15]. Thus, further studies are needed to elucidate how these complex bacterial interactions change within various soil conditions.

In addition to bacteria, worms also play a crucial role in soil functions and benefit soil ecosystems in many aspects. Worms can decompose plant litter and transform the litter into organic nutrients that benefit plants or other living organisms in the soil [16]. Burrowing of worms also changes the soil structure and can promote the ability of air and water to infiltrate the soil and thereby bring nutrients to the plant roots [17]. Through altering soil structure, worms also have the capacity to reduce the number of antibiotic resistance genes and heavy metals present in soil [18,19]. When worms convert organic materials into usable compost, vermicompost is created. The addition of vermicompost to soils provides higher aeration, porosity, drainage, and water holding capacity than traditional compost and thus supports plant growth [20,21]. Vermicompost alters the soil and root bacterial communities in both greenhouse and field conditions [22]. For example, Actinobacteria, an important bacterium for producing bioactive metabolites, was significantly more abundant in soil samples after 42 days of vermicomposting [23,24]. Vermicompost also presented greater bacterial richness than other types of compost [25].

Field soils are heavy and do not provide adequate water drainage and aeration for plant production in shallow containers. Thus, such soil is not used in greenhouse container production systems. The most common substrate used in horticultural seedling and container plant production, also called “the worldwide standard substrate”, is peat or peat-lite potting media [26,27]. Such media have sphagnum peat moss and horticultural vermiculite or perlite as their main components. Peat-lite potting medium is unique in that it has been shown to suppress plant diseases caused by soil-borne pathogens like *Pythium* spp. [28,29]. *Pythium ultimum* and *Pythium irregulare* cause seedling damping-off and root rot, which negatively impact overall plant health [30]. Compost samples mixed with sphagnum peat moss suppress *Pythium ultimum* damping-off in 64% of samples and *Pythium irregulare* damping-off in 67% of samples [29]. In general, nutrients are efficiently retained in peat moss, and decomposition rates are slower than in compost-based media. This is due to peat moss’s ability for high retention of water and organic matter [31]. Thus, peat moss-based plant media present distinct advantages in pathogen protection and nutrient retention for greenhouse container garden model systems.

By altering root mediums used in urban environments, soil biological activity and microbial diversity can be increased. Several studies have described the impact of vermicompost amendments on the rhizosphere and suggested it may be an effective growth alternative to peat [32,33]. However, there is no current literature exploring how vermicompost specifically interacts with peat-lite substrate to alter microbial communities and lettuce growth. Additionally, by using a novel greenhouse container garden model system, the microbial and nutritional changes observed may provide preliminary evidence for how to predict whether these growth conditions will affect human health. Herein, we investigate the effects of vermicompost amendments on the root media bacterial communities and harvest mass in a lettuce greenhouse container garden model system using seven root media/amendment conditions. We hypothesized that microbial communities of the root media and lettuce mass will be influenced by the addition of vermicompost in both peat and compost-based growing media.

## 2. Materials and Methods

### 2.1. Study Design and Setting

Three (4 gallon) containers of each of the seven growth media conditions were planted with lettuce seeds (cv. Tango) sown in the winter of 2018 in a temperature controlled (18–20 °C) glass glazed greenhouse in East Lansing, MI. Growth media consisted of either compost or peat-lite. The compost growth media (C) were made through thermophilic composting of a mixture of fall-collected municipal leaves, ramial wood chips from street or yard tree pruning, baled straw, baled hay, and kitchen preparation food residue including coffee grounds, and allowed to mature 12 months beyond completion of composting.

The peat-lite (PL) growth media were commercial growing media: PRO-MIX High Porosity (HP) with added mycorrhizae (Quakertown, PA, USA). Pro-Mix HP Biofungicide + Mycorrhizae is a high-porosity, peat-lite growing medium, ideal for water-sensitive crops, rooting cuttings and/or low-light growing conditions. Pro-Mix HP Biofungicide + Mycorrhizae features high perlite, peat-lite growing medium with high drainage capacity. The Pro-Mix HP Biofungicide + Mycorrhizae media also contain mycorrhizae mycorrhizal inoculum (*Glomus intraradices*) and biofungicide (*Bacillus pumilus* strain GHA-180).

The vermicompost amendment (V) was produced at the vermicompost research facility of Michigan State University. The vermicompost was made by initial thermophilic pre-composting a one to one by volume mixture of municipal collected fallen tree leaves mixed with cafeteria kitchen preparation vegetable/fruit scraps and coffee grounds for 3 to 4 weeks. The pre-composted material was then applied in 3-to-4-inch layers at 2-to-3-week time intervals on the 30-degree sloped leading edge of a 50′ long “wedge” windrow approximately 2 feet tall. The windrow and worms were maintained in an unheated passive solar polyethylene film covered greenhouse with full sun exposure in winter months (October–April) and with 90% shade fabric during summer months (May–September). Red wiggler composting worms (*Eisenia fetida*) were managed to allow horizontal movement into the pre-composted mixture with an active residence time of 6 to 8 weeks at a worm mass density of (1.5 to 3 pounds/square foot) and longer curing time at reduced worm mass density of 2 to 4 months. Final screened vermicompost averaged 2.5 to 3.5% N dry weight basis, pH 6.7 to 7.0, and Saturated Media Extract (SME) electrical conductivity (EC) of 8 to 12 mS/cm.

The vermicompost water extract treatment was prepared with screened (0.25″) worm compost at approximately 35 to 40% moisture content mixed in a 1:5 ratio by volume with tap water in a plastic bucket. The vermicompost was then extracted with mixing for 30 min followed by gravitational settling for 10 min, with the supernatant collected and applied as an irrigation.

The seven growth media conditions are categorized in Table 1. Treatments included: (1) the compost-based growing media with no additions (C), (2) C with a water extract (5:1) of vermicompost collected and applied as an irrigation at 2, 3 and 4 weeks (wks) of growth (CI), (3) C with vermicompost added at 10% by volume prior to planting (CV), and (4) C with worms (*Eisenia fetida*) and worm compost added to the surface of the growing media at 2 wks, 3 wks, and 4 wks of growth (CVW), (5) the commercial peat-lite soil-less growing media with no additions (PL), (6) PL with vermicompost added at 10% by volume prior to planting (PLV), and (7) PL with water extract (5:1) of vermicompost collected and applied as an irrigation at 2, 3 and 4 weeks (wks) of growth (PLI). Plants were watered on a regular schedule, with all containers being watered on the same day at the same time. Root zone media samples (n = 3 per condition) were collected just before lettuce harvest.

### 2.2. Lettuce Harvest

Lettuce (cv. Tango) grew in a temperature-controlled greenhouse on the Michigan State University main campus. Seeds were sown on 5 October 2018 and lettuce top growth was harvested initially four weeks later (5 November 2018) and then again on 13 December 2018. DNA was extracted from the growth media following the initial harvest on 5 November 2018. Following the second harvest on 13 December 2018, the lettuce was dried in a forced air oven to determine dry weight.

### 2.3. DNA Extraction and 16S rRNA Gene PCR Amplification

DNA was extracted from the growth media samples using the MoBio Powersoil^@^ DNA Isolation kit (Qiagen MoBio, Carlsbad, CA, USA), using the Human Microbiome Project’s method (HMP) [34] with a few alterations: samples were centrifuged for 2 min after incubation in C3 solution (from the kit) for 5 min, and DNA was eluted from the filter membrane with 50 μL of 55 °C low-EDTA buffer (IDT, Coralville, IA, USA).

The V4 region of the 16S rRNA gene was amplified using barcoded primers, and sequences were processed using mothur version 1.44.2 [35]. The Schloss lab primers (500B-700A) were used for amplification [36]. These primers were ordered from IDT (Coralville, IA, USA). PCR amplification procedure followed the mothur wet lab documentation [35]. A final reaction volume of 20 μL with at most 10 ng of template DNA, primer pairs and Accumprime Pfx Supermix (ThermoFisher, Waltham, MA, USA) was used. The PCR reactions were performed in triplicate and amplified using a thermocycler. A negative control without template DNA was included to control for non-specific amplification. Thermocycler conditions were as follows: 1× (95 °C for 2 min); 30× (95 °C for 20 s, 55 °C for 15 s, 72 °C for 5 min); 10 min for 72 °C. The reaction products were checked by agarose gel electrophoresis on a 1% agarose gel using 1× TBE buffer at 200 V for 30 min. Successful PCR triplicate products were pooled and cleaned with Agencourt AMPure XP (Beckman Coulter, Brea, CA, USA) with a few changes to the protocol: PCR products were purified by 0.7× AMPure XP and DNA was eluted using 20 μL of low EDTA TE buffer (IDT, Coralville, IA, USA). After purification, the 16S rRNA DNA concentrations were determined by Quant-IT dsDNA assay kit (Invitrogen, Carlsbad, CA, USA). Purified DNA amplicons were pooled and the quality of the resulting amplicons was checked using an Agilent 2100 Bioanalyzer with the High Sensitivity DNA Chip (Agilent (Santa Clara, CA, USA), 5067–4626). Pooled amplicons were diluted to 2.5 ng/μL. Then an equal amount (ng) of each sample was pooled for sequencing. The Michigan State University Research Technology Support Facility Genomics Core conducted paired-end 250 base-pair sequencing on the Illumina MiSeq platform using V2 chemistry.

### 2.4. Processing and Analysis of 16S rRNA Gene Sequencing Data

16S rRNA sequences were processed using mothur, following the mothur SOP [36]. Taxonomy was assigned to operational taxonomic units (OTU) by phylotype using the SILVA database (release 128) [37]. Samples were rarefied to 15,000 reads per sample before further analysis and adequate microbial community coverage was confirmed by rarefaction curves.

### 2.5. Statistical Analyses

Data were analyzed using R version 4.2.2 [38]. Alpha diversity (Chao 1, inverse Simpson and Shannon indices) was calculated for each of the seven growing conditions in R using the vegan package version 2.5-4 [39]. For Alpha diversity analysis, Kruskal–Wallis with Dunn’s test or one-way ANOVA with Tukey HSD were applied to compare the data at both the genus and phylum levels based on the normality of the data (Table 2). For Beta diversity, the dissimilarities across the seven planting conditions, between all C media and all P media conditions, within C media conditions, and within P media conditions were determined by Sorensen (community membership) and Bray–Curtis (community composition/structure) distance metrics using permutational multivariate analysis of variance (PERMANOVA) and permutation of dispersion (PERMDISP) and visualized using principal coordinate analysis (PCoA) using vegan package (Table 2). PERMANOVA was performed to test differences across groups. PERMDISP was used to test for differences in sample dispersion. Individual taxa were compared across groups using a negative binomial model in the MASS package 7.3-52 [40] on the taxa that composed >1% abundance on average. For the lettuce growth data, ANOVA with Tukey HSD was applied to compare the fresh weight, dry weight, and the percentage of the dry weight of lettuce after confirming normality using the Shapiro–Wilk test. The Benjamini–Hochberg adjustment was used for false discovery rate correction. *p*-values less than 0.05 were considered significant.

## 3. Results

### 3.1. Alpha Diversity

**Figure 1:**
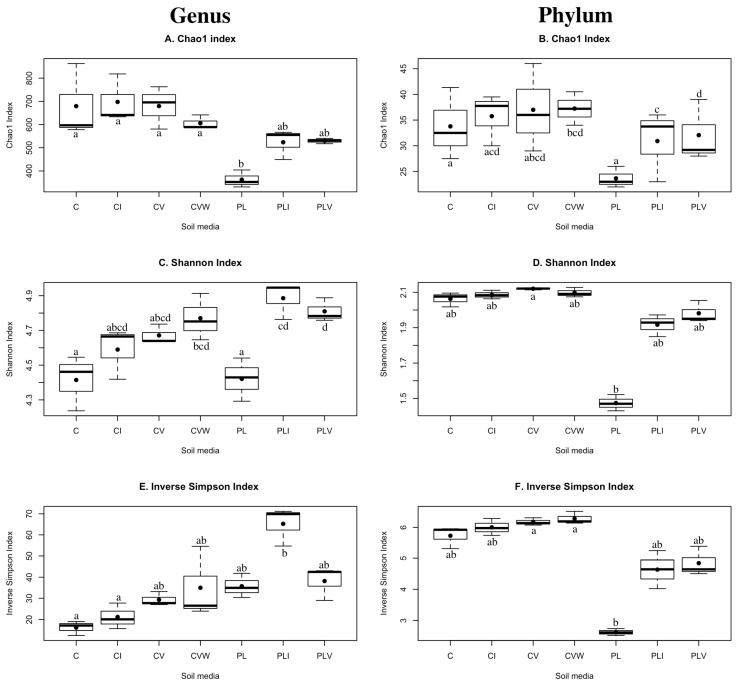
Alpha diversity of the bacterial communities in each the seven root media/amendment conditions. Genus-level alpha diversity is presented in Panels (**A**,**C**,**E**), and phylum-level alpha diversity is presented in panels (**B**,**D**,**F**). The richness (Chao 1) of each community is represented in panels (**A**,**B**). Both the richness and abundance (Shannon, sensitive to rare taxa) of each community is indicated in panels (**C**,**D**). The diversity (Inverse Simpson, more weight to dominant taxa) of each community is indicated in panels (**E**,**F**). Soil treatment conditions include (C) for Compost-based growing media with no additions, (CI) for C with a water extract (5:1) of vermicompost collected and applied as an irrigation at 2, 3 and 4 weeks (wks) of growth, (CV) C with vermicompost added at 10% by volume prior to planting, (CVW) C with worms and worm compost added to the surface of the growing media at 2 wks, 3 wks, and 4 wks of growth, (PL) a commercial peat-lite (PRO-MIX, Quakertown, PA, USA) soil-less growing media with no additions, (PLI) PL with water extract (5:1) of vermicompost collected and applied as an irrigation at 2, 3 and 4 weeks (wks) of growth, (PLV) PL with vermicompost added at 10% by volume prior to planting. The line across the boxplot presents the median of the data. The whiskers of the boxplot indicate the interquartile range. Unique letters above or below the boxes indicate significant differences (*p* < 0.05) between the groups.

At the genus level, the bacterial communities of PL had similar richness as PLI and PLV but significantly lower richness than all four conditions with C growing media (Chao 1, Figure 1A). However, when measured using the Shannon index, bacterial diversity, at the genus level, in PL treatment condition was similar to all C media conditions with the exception of CVW, but lower than that of the PLI and PLV treatment conditions (Figure 1C). Moreover, when measured by the Inverse Simpson index, the PL treatment condition had similar diversity, at the genus level, to all other treatment conditions (inverse Simpson, Figure 1E). At the phylum level, the richness of bacterial communities in PL treatment condition was significantly lower than that of the PLI and PLV (Chao 1, Figure 1B). Bacterial diversity in the PL treatment condition was lower than that of the other six groups but only reached statistical significance when compared to the CV treatment condition (Shannon, Figure 1D). This was similar when bacterial diversity was assessed by the Inverse Simpson index, but, in this case, the PL treatment condition had significantly lower diversity than either the CV or CVW treatment conditions (Inverse Simpson, Figure 1F).

### 3.2. Beta Diversity

**Figure 2:**
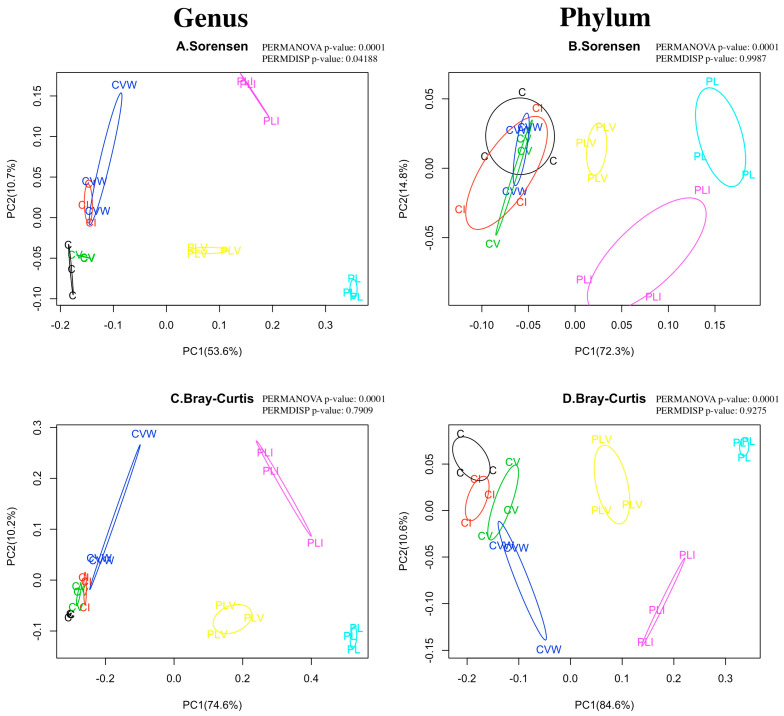
Beta diversity comparison of all seven root media/amendment conditions by genus (**A**,**C**) and phylum (**B**,**D**). The Sorensen metric accounts for presence/absence (**A**,**B**), whereas the Bray–Curtis beta-diversity metric accounts for abundance (**C**,**D**). Growing media treatment conditions include (C) for Compost-based growing media with no additions, (CI) for C with a water extract (5:1) of vermicompost collected and applied as an irrigation at 2, 3 and 4 weeks (wks) of growth, (CV) C with vermicompost added at 10% by volume prior to planting, (CVW) C with worms and worm compost added to the surface of the growing media at 2 wks, 3 wks, and 4 wks of growth, (PL) a commercial peat-lite (PRO-MIX, Quakertown, PA, USA) soil-less growing media with no additions, (PLI) PL with water extract (5:1) of vermicompost collected and applied as an irrigation at 2, 3 and 4 weeks (wks) of growth, (PLV) PL with vermicompost added at 10% by volume prior to planting. The percentages on the axes indicate the proportion of variance explained by each principal coordinate axis based on the distance matrix.

At the genus level, root media bacterial communities from the seven growth media conditions differed in membership (Sorensen; Figure 2A) and composition (Bray–Curtis; Figure 2C). All groups containing PL had distinct membership and composition from the C groups at both the genus (Figure 2A and Figure S1A) and the phylum (Figure 2B and Figure S1B) levels as indicated by strong separation across PC1 (Figure 2 and Figure S1). Whereas the 3 PL treatment groups (PL, PLI, PLV) differed in composition at both the genus (Figure 2A) and phylum (Figure 2B) level, this was not the case for the C treatment groups. All C groups (C, CV, CI, CVW) overlapped in phyla membership (Figure 2B and Figure S2B). At the genus level, CVW and CI overlapped in membership, in addition, C and CV overlapped in membership with each other but had distinct membership from CVW and CI (Figure 2A and Figure S2A). When taking into account both membership and abundance, i.e., composition, similar trends were observed. All C groups were distinct from all PL groups at the genus (Figure 2C and Figure S1C) and phylum (Figure 2D and Figure S1D) levels as evidenced by the spread across PC1 where all C groups were to the left of zero along PC1 and all PL groups were to the right of zero. Bacterial communities in C media have higher similarity than those of PL media for both membership (Sorensen; Figure 2A,B) and composition (Bray–Curtis; Figure 2C,D).

Within C media, when analyzed in the absence of P treatment groups, CVW and CI had somewhat similar membership distinct from that of CV and C (Sorensen; Figure S2A) at the genus level. The composition of the four C groups was more distinct at the genus level with only some overlap between CI and CV (Bray–Curtis; Figure S2C). At the phylum level, membership in the C groups had distinct centroids but were overlapping (Sorensen, Figure S2B) whereas the composition was distinct with little overlap (Figure S2D). The three treatments in P media differed by membership (Sorensen; Figure S3A,B) and composition (Bray–Curtis; Figure S3C,D) at both the genus and phylum levels.

### 3.3. Taxa Specific Differences

#### 3.3.1. Individual Microbial Differences Between PL (PL, PLI, PLV) and C (C, CI, CV, CVW) Media

**Figure 3:**
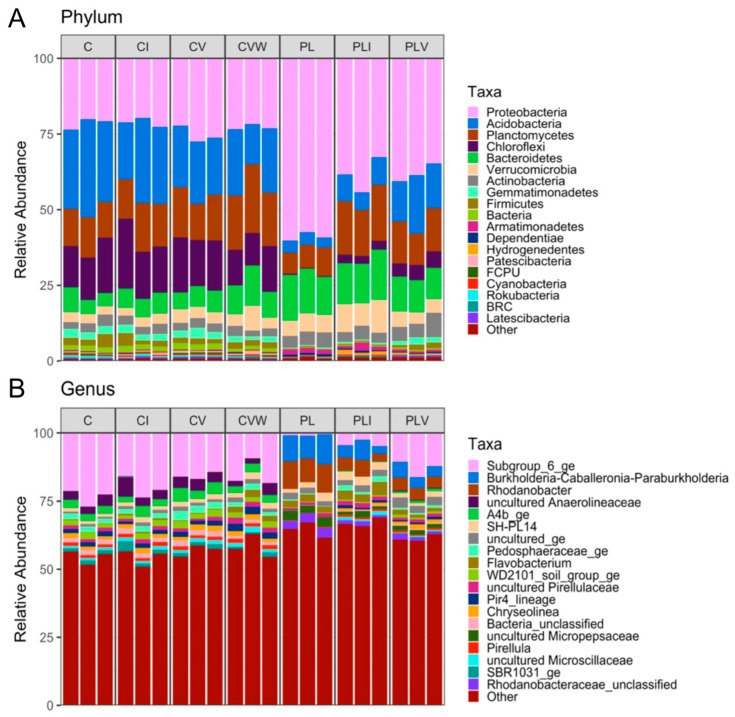
Bar chart of the phyla (**A**) and genera (**B**) contained in each treatment. N = 3 per treatment. Growing media treatment conditions include (C) for Compost-based growing media with no additions, (CI) for C with a water extract (5:1) of vermicompost collected and applied as an irrigation at 2, 3 and 4 weeks (wks) of growth, (CV) C with vermicompost added at 10% by volume prior to planting, (CVW) C with worms and worm compost added to the surface of the growing media at 2 wks, 3 wks, and 4 wks of growth, (PL) a commercial peat-lite (PRO-MIX, Quakertown, PA, USA) soil-less growing media with no additions, (PLI) PL with water extract (5:1) of vermicompost collected and applied as an irrigation at 2, 3 and 4 weeks (wks) of growth, (PLV) PL with vermicompost added at 10% by volume prior to planting.

At the phylum level, *Proteobacteria*, *Actinobacteria*, *Bacteroidetes*, and *Verrucomicrobia* were more abundant in PL media than C media (Table 2), whereas *Chloroflexi*, *Firmicutes*, *Acidobacteria*, and *Gemmatimonadetes* were more abundant in C media (Table 3). Interestingly, the relative abundance of *Planctomycetes* was similar in C media and PL media (Table 3). Differences between C and PL at the genus level were mainly driven by unclassified or low abundance bacteria (Table 3, Figure 3). C media and PL media contained a similar relative abundance of uncultured *Pirellulaceae* (Table 3). PL media had higher relative abundance of *Flavobacterium*, *SH PL14*, uncultured *Micropepsaceae*, *Burkholderia*-*Caballeronia*-*Paraburkholderia*, and *Rhodanobacter* than C media (Table 3).

#### 3.3.2. Individual Microbial Differences Among the Seven Root Media/Amendment Conditions

At phylum level, PL had the highest abundance of *Proteobacteria*, but lowest abundance of *Planctomycetes*, *Acidobacteria*, and *Chlorflexi* among all 7 growth conditions (Table 4). PL, PLI, and PLV had significantly different abundance of *Planctomycetes*, *Acidobacteria* and *Chlorflexi* regardless of the C groups (Table S1). Addition of just vermicompost water extract inoculate to the PL media (PLI) increased *Planctomycetes*, *Acidobacteria*, *Chloroflexi*, and *Verrucomicrobia* but decreased *Proteobacteria* (Table S1). PLI and PLV had similar abundances of *Proteobacteria* (Table S1). Moreover, the overall composition of the bacterial communities within the C growing media was different regardless of vermicompost treatment except for *Actinobacteria* and *Firmicutes* (Table S2). The addition of vermicompost and worms (CVW) increased abundance of *Planctomycetes* and *Bacteroidetes*, and decreased abundance of *Chlorflexi* in compost-based media (Table S2).

At the genus level, PL had the lowest abundance of *Subgroup 6 ge*, Bacteria unclassified, *WD2101 soil group ge*, *Pedosphaeraceae ge*, and *Pir4* lineage, along with the highest abundance of uncultured *Micropepsaceae*, *Burkholderia-Caballeronia-Paraburkholderia*, and *Rhodanobacter* among all 7 growth conditions (Table 5). Among PL groups, abundance of *Subgroup 6 ge*, uncultured *Pirellulaceae* and *Flavobacterium* was higher after the addition of vermicompost irrigation (PLI) (Table S3). *Subgroup 6 ge* in PLV had a higher abundance than in PL and PLI media (Table S3). C, CI, CV, and CVW had similar abundance of *Chryseolinea*, *Pirellula*, uncultured *Anaerolineaceae*, and *WD2101 soil group ge* (Table S4). Addition of vermicompost and worms (CVW) decreased Bacteria unclassified, but increased *Pir4 lineage* (Table S4).

### 3.4. Lettuce Weight

**Figure 4:**
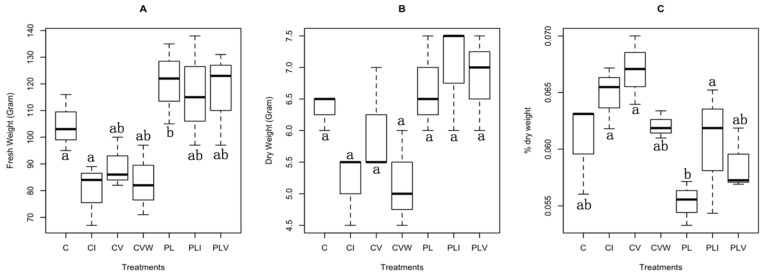
Fresh weight (**A**), dry weight (**B**), and percentage of dry weight (**C**) of lettuce grown using the seven root media/amendment conditions. Growing media treatment conditions include (C) for Compost-based growing media with no additions, (CI) for C with a water extract (5:1) of vermicompost collected and applied as an irrigation at 2, 3 and 4 weeks (wks) of growth, (CV) C with vermicompost added at 10% by volume prior to planting, (CVW) C with worms and worm compost added to the surface of the growing media at 2 wks, 3 wks, and 4 wks of growth, (PL) a commercial peat-lite (PRO-MIX, Quakertown, PA, USA) soil-less growing media with no additions, (PLI) PL with water extract (5:1) of vermicompost collected and applied as an irrigation at 2, 3 and 4 weeks (wks) of growth, (PLV) PL with vermicompost added at 10% by volume prior to planting. The line across the boxplot presents the median of the data. The whiskers of the boxplot indicate the interquartile range. Unique letters above or below the boxes indicate significant differences (*p* < 0.05) between the groups.

The fresh weight of harvested lettuce was similar for all growing conditions with the exception that lettuce grown in PL had higher fresh weight than lettuce harvested from C or CI growth media (Figure 4A). Dry weights were similar across all treatments (Figure 4B). When normalized by wet weight, the percentage dry weight, an inverse measure of water content and an estimate of nutrient density, of lettuce harvested from CI, CV, and PLI was higher than that of lettuce harvested from PL (Figure 4C). Vermicompost-based amendments (I, V) to PL and C growing media insignificantly increased lettuce production on a percent dry weight basis (Figure 4C).

## 4. Discussion

This study establishes a low-cost, replicable protocol for studying the impacts of compost-based (C) and peat-lite (PL) growth media with vermicompost-related amendments in a greenhouse container garden model system. We identified the impacts of PRO-MIX HP + mycorrhizae (PL) and compost media (C), both with various vermicompost amendments, on root zone bacterial communities. This data provides strong evidence that bacterial diversity in the soil of a greenhouse container model is altered by different root media amendments. The compost-based and peat-lite growing media had distinct bacterial communities at both the phylum and genus levels. Vermicompost amendments to PL growing media increased Shannon diversity (Figure 1C,D). Furthermore, the addition of vermicompost-based amendments to PL reduced, but did not eliminate, the dissimilarity between PL bacterial communities and C bacterial communities (Figure 2). A vermicompost-based amendment (PLI) to PL increased lettuce production on a percent dry weight basis (Figure 4C), bringing the percent dry weight to the same levels as observed for all compost-based growth media conditions.

With vermicompost added, the richness of growth media bacterial communities was not significantly changed in either PL or C growing media at the genus level (Figure 1A). However, diversity was increased by the addition of vermicompost among PL groups (Figure 1C). This finding aligns with previous literature which found that higher bacterial richness and diversity was demonstrated in the vermicompost than other types of compost [41,42]. At the phylum level, vermicompost increased the richness of bacteria in PL growth media (Figure 1B). However, the diversity of bacterial communities at the phylum level was similar across growing conditions (Figure 1D,F). Vermiculture inoculant had the same effects on alpha diversity as vermiculture itself in both compost-based and peat-lite based growth media (Figure 1C–F). In turn, the addition of vermicompost amendments had a stronger impact in PL than in C growth media. Thus, vermicompost amendments are typically an effective method to increase the alpha diversity of PL growth media.

Emerging evidence has continued to support the idea that interactions between plant growth and soil microbial diversity significantly affect crops. Plant roots are in close contact with a complex ecosystem of soil bacteria that can be utilized to improve the quality and nutritional value of seeds, thereby increasing food productivity and quality [43,44,45,46]. Here, lettuce grown under the CI condition had a higher percentage of dry weight than that of lettuce grown under the PL condition (Figure 4C). A higher percentage of dry weight is an indicator of nutritional density, as it indicates that less of the harvest weight of the lettuce is water. Interestingly, PL also had the lowest bacterial richness compared to the other 6 treatment conditions (Figure 1A). Increased activity from soil bacteria can increase nitrogen mineralization, nutrient cycling, and plant growth [43,47]. At the phylum level, CI and CV had a significantly higher abundance of *Planctomycetes*, *Acidobacteria*, *Chloroflexi*, *Firmicutes*, and *Gemmatimonadetes* compared to PL treatment conditions. Previous research has linked each of these bacterial taxa groups to a variety of important functions in the soil microbiome that assist with plant growth. Phyla including *Chloroflexi*, *Firmicutes*, *Acidobacteria* and *Gemmatimonadetes* are important in maintaining fixed carbon levels in soil carbon reservoirs [48]. Active cellulose metabolism is also carried out by members of phylum *Chloroflexi* and *Planctomycetes*, which contributes to nitrogen fixation and anaerobic ammonia oxidation [48,49]. As previously mentioned, the PL group had the lowest bacterial richness, and such loss of microbial diversity has been associated with impaired nitrogen-cycle functioning, which negatively impacts plant growth and nutrient uptake [50,51]. We hypothesize that without an adequate number of bacteria that contribute to nutrient availability, such as *Actinobacteria*, *Acidobacteria*, and *Gemmatimonadetes*, the PL growth condition may have been unable to adequately utilize available micronutrients. The PL growth media also had the lowest percent dry weight, corresponding to low nutrient density. An important feature to note about peat-based growing media is that there is limited nutrient availability, especially nitrogen [52,53]. Previous experiments have shown that adding vermicompost, which has greater nitrogen and phosphorous availability, can increase plant size and lettuce production compared to just a peat based medium [54,55]. Therefore, it is important to consider that the correlation between nutrient density and microbial activity may be mediated by the available micronutrients provided by each compost type.

Soil biodiversity is of major importance to ecosystem health. Soil biodiversity affects the regulation of pests and pathogens, along with nutrient release. Growing evidence demonstrates that ecosystems with a high level of biodiversity might provide high-level services for the ecosystem. High biodiversity has been shown to provide more tolerance to natural and anthropogenic disturbances [56,57]. Not only can biodiversity impact ecosystems, but biodiversity can also have vital positive effects on human health. Both the human and soil microbiomes are closely linked and share many similar characteristics. The human and soil microbiomes share similar bacterial phyla (*Firmicutes*, *Bacteroidetes*, *Proteobacteria*, *Actinobacteria*) and are composed of open systems that depend on unique oxygen, water and pH gradients [58]. Humans can also be in close contact with soil and the microorganisms in it during their everyday lives. As humans consume different fruits and vegetables, the microbes associated with those fruits and vegetables are passed into the human gut, interact with the human gut microbiome, and thereby potentially affect human health. The human gut microbiome potentially can also gain new bacteria from the consumption of raw plant material with soil particles attached [1,59]. Due to the role of the soil microbiome in the environment and human health, research regarding the connection between food system microbiomes and human microbiomes should continue.

This study establishes initial data to support further exploration of how vermicompost, combined with other peat-lite and compost substrates, can alter the rhizosphere in a greenhouse container garden model. Future studies should aim to detect whether the consumption of lettuce grown in greenhouse container conditions can also impact human microbiomes. Such studies will determine if we can detect soil bacteria in the stool of humans who consume the lettuce grown under the various root media conditions. We hypothesize that the bacteria in or on the lettuce will be transmissible to and detectable in humans, which has been shown to be feasible [1,60], but is yet unproven.

However, the experiments presented herein must be reproduced on a larger scale—more experimental replicates and more soil samples per treatment in each replicate as well as more careful assessment of nitrogen content, pH, and EC of each growth media. Furthermore, bacterial communities in greenhouses likely differ from those in the natural environment [61]. Therefore, the current experimental design can apply to smaller scale controlled agricultural farming techniques but has limited generalizability for the average home container garden. Thus, the repetition of these experiments in an outdoor setting, both in controlled experimental situations as well as at participant homes, could be beneficial. Additionally, comparing results from greenhouse container garden model system microbiota communities in urban versus suburban or rural areas could provide valuable insight regarding the effects of the surrounding environment on container growth media bacterial communities. A question that remains is whether the variability of compost media and vermicompost production methods can be managed to provide reproducible results. A longer term goal of this research is to identify growing media treatments that can reproducibly alter the microbiome of harvested lettuce and possibly the human microbiome.

## 5. Conclusions

In conclusion, the type of growth media and vermicompost amendments impacted the diversity and abundance of growth media bacteria present in a greenhouse container garden model system. Addition of a vermicompost-based amendment (PLI) to PL increased lettuce production on a percent dry weight basis. The PL medium used herein is widely available in the United States and may provide similar results for other researchers. Future research should address the impact that consumption of produce grown on these amended media can have on the composition and function of the crop endophytes and/or the human gut microbiota. 

## Figures and Tables

**Table 1:** Composition of the seven root media/amendment conditions used in the greenhouse container garden model system.

Treatment Abbreviation	Growing-Media Composition
C	Compost-based growing medium with no additions
CI	Compost-based growing medium with water extract (5:1) of vermicompost collected and applied as an irrigation at 2, 3, and 4 weeks of growth
CV	Compost-based growing medium with vermicompost added at 10% by volume prior to planting
CVW	Compost-based growing medium with worms and worm compost added to the surface of the growing media at 2, 3, and 4 weeks of growth
PL	Peat-lite soil-less growing medium with no additions
PLI	Peat-lite with water extract (5:1) of vermicompost collected and applied as an irrigation at 2, 3, and 4 weeks of growth
PLV	Peat-lite with vermicompost added at 10% by volume prior to planting

**Table 2:** Statistical tests used to measure each soil diversity metric.

Soil Diversity Metric	Statistical Tests
Data Normality	Shapiro–Wilk
Alpha Diversity	Kruskal–Wallis with Dunn’s test or one-way ANOVA with Tukey HSD
Beta Diversity	PERMANOVA and PERMDISP
Individual Taxa Differences	Negative Binomial model
Lettuce Growth Yield	ANOVA with Tukey HSD with The Benjamini–Hochberg adjustment

**Table 3:** Taxa relative abundance in C (combined C, CI, CV, CVW) and PL (combined PL, PLI, PLV) growing media at the phylum and genus levels.

**Taxa—Phylum Level**	**Overall**	**C Media**	**PL Media**	***p*-Value**
*Proteobacteria*	32.3 ± 13.4	22.6 ± 2.3	45.1 ± 10.9	<0.0001
*Chloroflexi*	10.1 ± 7.1	15.6 ± 3.2	2.7 ± 2	<0.0001
*Firmicutes*	1.6 ± 1.2	2.2 ± 1	0.7 ± 0.7	<0.0001
*Acidobacteria*	17 ± 8.6	22.9 ± 5.1	9.2 ± 5.6	<0.0001
*Gemmatimonadetes*	1.8 ± 1	2.4 ± 0.6	0.9 ± 0.7	<0.0001
*Actinobacteria*	3.6 ± 1.3	2.9 ± 0.5	4.6 ± 1.4	<0.0001
*Bacteroidetes*	9.8 ± 3.7	7.3 ± 2.4	13.2 ± 2.1	<0.0001
*Planctomycetes*	14.1 ± 3.8	15.2 ± 3.2	12.7 ± 4.1	0.1000
*Verrucomicrobia*	4.9 ± 2.3	3.8 ± 1.5	6.4 ± 2.3	0.0002
**Taxa—Genus Level**	**Overall**	**C Media**	**PL Media**	***p*-Value**
*Subgroup 6 ge*	13.1 ± 8.2	18.6 ± 4.7	5.9 ± 5.6	0.0004
*Bacteria_unclassified*	1.1 ± 0.8	1.7 ± 0.4	0.3 ± 0.2	<0.0001
*WD2101 soil group ge*	1.4 ± 0.9	2 ± 0.5	0.5 ± 0.3	<0.0001
*A4b ge*	2 ± 1.6	3.2 ± 0.9	0.4 ± 0.4	<0.0001
*Pedosphaeraceae ge*	1.6 ± 0.8	2.1 ± 0.6	0.9 ± 0.7	0.0001
*uncultured Pirellulaceae*	1.4 ± 0.4	1.4 ± 0.4	1.2 ± 0.3	0.2000
*Flavobacterium*	1.5 ± 1.3	0.8 ± 0.9	2.5 ± 1.1	0.0020
*Pir4_lineage*	1.3 ± 0.8	1.8 ± 0.5	0.6 ± 0.4	<0.0001
*uncultured Anaerolineaceae*	2.1 ± 2.1	3.7 ± 1.4	0.1 ± 0.1	<0.0001
*SH PL14*	1.9 ± 0.7	1.6 ± 0.5	2.2 ± 0.8	0.0200
*uncultured Micropepsaceae*	1 ± 1.3	0.1 ± 0	2.3 ± 1	<0.0001
*Chryseolinea*	1.2 ± 0.7	1.6 ± 0.4	0.6 ± 0.6	<0.0001
*Burkholderia Caballeronia Paraburkholderia*	2.7 ± 3.7	0 ± 0.1	6.3 ± 2.8	<0.0001
*Rhodanobacter*	2.7 ± 3.6	0.1 ± 0.1	6.2 ± 2.8	<0.0001

Values reported as mean ± SD. *p*-values were Benjamini–Hochberg corrected.

**Table 4:** Taxa relative abundance in the seven root media/amendment conditions at the phylum level.

Taxa	Overall	C	CI	CV	CVW	PL	PLI	PLV	*p*-Value
*Proteobacteria*	32.3 ± 13.4	21.4 ± 1.9 ^a^	21.1 ± 1.5 ^a^	25.3 ± 2.7 ^b^	22.7 ± 0.9 ^ab^	58.9 ± 1.4 ^c^	38.4 ± 5.8 ^d^	37.9 ± 3 ^d^	<0.0001
*Planctomycetes*	14.1 ± 3.8	12.5 ± 0.8 ^ae^	14.4 ± 1.5 ^ade^	14.5 ± 2.4 ^ade^	19.4 ± 2.8 ^bd^	8 ± 1.3 ^c^	17.1 ± 1.7 ^d^	12.8 ± 2.1 ^e^	<0.0001
*Acidobacteria*	17 ± 8.6	28.5 ± 3.5 ^a^	24.2 ± 4.8 ^abc^	19.9 ± 0.9 ^bcf^	18.8 ± 4.8 ^cf^	3.8 ± 0.5 ^d^	8 ± 1.8 ^e^	15.8 ± 3.1 ^f^	<0.0001
*Chloroflexi*	10.1 ± 7.1	15.3 ± 2.6 ^a^	17.9 ± 4.4 ^a^	16.7 ± 1.4 ^a^	12.5 ± 2.3 ^b^	0.3 ± 0.1 ^c^	2.7 ± 0.2 ^d^	5 ± 0.6 ^e^	<0.0001
*Bacteroidetes*	9.8 ± 3.7	5.8 ± 2.1 ^a^	6.5 ± 0.5 ^a^	6.6 ± 0.9 ^a^	10.5 ± 2.5 ^be^	14.2 ± 1.4 ^cd^	14.4 ± 1.9 ^d^	10.9 ± 0.6 ^e^	<0.0001
*Actinobacteria*	3.6 ± 1.3	3 ± 0.8 ^ac^	3 ± 0.4 ^ac^	2.9 ± 0.3 ^ac^	2.7 ± 0.7 ^a^	4.5 ± 0.5 ^bcd^	3.7 ± 0.7 ^c^	5.7 ± 2 ^d^	<0.0001
*Verrucomicrobia*	4.9 ± 2.3	3 ± 0.3 ^a^	3 ± 0.4 ^a^	3.9 ± 0.5 ^abe^	5.2 ± 2.6 ^bce^	5.6 ± 0.5 ^ce^	9.2 ± 1.7 ^d^	4.4 ± 0.7 ^e^	<0.0001
*Firmicutes*	1.6 ± 1.2	2.9 ± 1.3 ^a^	2.4 ± 1.6 ^ad^	1.7 ± 0.3 ^ad^	2 ± 0.2 ^ad^	0.2 ± 0.1 ^bc^	0.3 ± 0 ^c^	1.5 ± 0.6 ^d^	<0.0001
*Gemmatimonadetes*	1.8 ± 1	2.7 ± 0.3 ^a^	2.4 ± 0.4 ^ad^	2.8 ± 0.7 ^ad^	1.7 ± 0.5 ^ad^	0.4 ± 0.1 ^bc^	0.5 ± 0.1 ^c^	1.8 ± 0.5 ^d^	<0.0001

Values reported as mean ± SD. Values in a row that do not contain the same superscript are significantly different, *p* < 0.05. *p*-values were Benjamini–Hochberg corrected.

**Table 5:** Taxa relative abundance in the seven root media/amendment conditions at the genus level.

Taxa Name	Overall	C	CI	CV	CVW	PL	PLI	PLV	*p*-Value
*Subgroup 6 ge*	13.1 ± 8.2	23.6 ± 3 ^a^	20 ± 4 ^ab^	15.7 ± 1.3 ^abe^	15 ± 5 ^be^	0.8 ± 0.2 ^c^	3.9 ± 1.3 ^d^	12.9 ± 2.9 ^e^	<0.0001
*Bacteria unclassified*	1.1 ± 0.7	1.7 ± 0.2 ^a^	1.8 ± 0.3 ^a^	2 ± 0.1 ^a^	1.2 ± 0.1 ^b^	0.1 ± 0.1 ^c^	0.3 ± 0 ^d^	0.5 ± 0.1 ^e^	<0.0001
*WD2101 soil group ge*	1.4 ± 0.9	1.9 ± 0.5 ^a^	1.9 ± 0.6 ^a^	2.4 ± 0.5 ^a^	2 ± 0.3 ^a^	0.3 ± 0 ^b^	0.5 ± 0.1 ^c^	0.8 ± 0.3 ^d^	<0.0001
*A4b ge*	2 ± 1.6	2.9 ± 0.2 ^ab^	2.9 ± 1 ^ab^	4.2 ± 1 ^a^	2.9 ± 0.4 ^b^	0 ± 0 ^abcd^	0.2 ± 0.2 ^c^	0.9 ± 0.2 ^d^	<0.0001
*Pedosphaeraceae ge*	1.6 ± 0.8	1.9 ± 0.2 ^abd^	1.9 ± 0.2 ^abd^	2.8 ± 0.5 ^a^	1.7 ± 0.5 ^bd^	0.4 ± 0.1 ^c^	1.7 ± 0.6 ^d^	0.7 ± 0.2 ^e^	<0.0001
*uncultured Pirellulaceae*	1.4 ± 0.4	1.3 ± 0.2 ^acde^	1.4 ± 0.3 ^ade^	1.2 ± 0.4 ^ace^	1.9 ± 0.3 ^bd^	1 ± 0.4 ^ce^	1.6 ± 0.2 ^d^	1.1 ± 0.1 ^e^	<0.0001
*Flavobacterium*	1.5 ± 1.3	0.2 ± 0.1 ^ac^	0.6 ± 0.1 ^b^	0.2 ± 0 ^c^	2.3 ± 0.4 ^de^	2.4 ± 0.3 ^e^	3.6 ± 1.1 ^f^	1.4 ± 0.4 ^g^	<0.0001
*Pir4 lineage*	1.3 ± 0.8	1.7 ± 0.1 ^a^	1.3 ± 0.3 ^a^	1.7 ± 0.2 ^a^	2.6 ± 0.3 ^b^	0.1 ± 0 ^c^	0.8 ± 0.2 ^de^	0.7 ± 0.3 ^e^	<0.0001
*uncultured Anaerolineaceae*	2.1 ± 2.1	3.4 ± 0.8 ^a^	4.5 ± 2.2 ^a^	4 ± 0.3 ^a^	2.7 ± 1.4 ^a^	0 ± 0 ^abc^	0 ± 0 ^b^	0.3 ± 0 ^c^	<0.0001
*SH PL14*	1.9 ± 0.7	1.1 ± 0.1 ^ae^	1.5 ± 0.4 ^abce^	1.6 ± 0.3 ^abce^	2.1 ± 0.4 ^bce^	2.2 ± 0.9 ^ce^	3 ± 0.2 ^d^	1.5 ± 0.5 ^e^	<0.0001
*uncultured Micropepsaceae*	1 ± 1.3	0.1 ± 0.1 ^a^	0.1 ± 0 ^a^	0.1 ± 0.1 ^a^	0.1 ± 0 ^a^	3.4 ± 0.5 ^b^	1.7 ± 0.8 ^cd^	1.8 ± 0.2 ^d^	<0.0001
*Chryseolinea*	1.2 ± 0.7	1.4 ± 0.4 ^a^	1.6 ± 0.4 ^a^	1.8 ± 0.5 ^a^	1.6 ± 0.4 ^a^	0 ± 0 ^abc^	0.7 ± 0.4 ^b^	1.2 ± 0.4 ^c^	<0.0001
*Burkholderia Caballeronia Paraburkholderia*	2.7 ± 3.7	0 ± 0 ^abcd^	0.1 ± 0.2 ^a^	0 ± 0 ^a^	0 ± 0 ^abcd^	9.6 ± 1.3 ^b^	4.9 ± 2.3 ^cd^	4.6 ± 1.2 ^d^	<0.0001
*Rhodanobacter*	2.7 ± 3.6	0 ± 0 ^abcde^	0.1 ± 0.1 ^a^	0.1 ± 0 ^ab^	0 ± 0 ^b^	9.8 ± 1 ^c^	4.5 ± 1.6 ^de^	4.5 ± 0.3 ^e^	<0.0001

Values reported as mean ± SD. Values in a row that do not contain the same superscript are significantly different, *p* < 0.05. *p*-values were Benjamini–Hochberg corrected.

## Data Availability

The raw data supporting the conclusions of this article will be made available by the authors on request.

## Supplementary Materials

The following supporting information can be downloaded at: https://www.mdpi.com/article/10.3390/microorganisms13081885/s1, Figure S1: Comparison of the beta diversity of the two base growing media conditions by genus (A,C) and phylum (B,D); Figure S2: Comparison of the beta diversity of the growth media conditions within the compost-based growth media by phylum (A,B) and genus (C,D); Figure S3: Comparison of the beta diversity of the growth media conditions within the Peat Lite-based growth media by phylum (A,B) and genus (C,D); Table S1: Individual taxa comparison in PL, PLI, and PLV at the phylum level.; Table S2: Individual taxa comparison in C, CI, CV, and CVW at the phylum level; Table S3: Individual taxa comparison in PL, PLI, and PLV at the genus level; Table S4: Individual taxa comparison in C, CI, CV, and CVW at genus level.
