# Supplementary material for: Influence of Two Root Media and Three Vermicompost Amendments on Bacterial Communities in a Greenhouse Container Garden Model System
Source: Microorganisms. 2025 Aug 13;13(8):1885. doi: 10.3390/microorganisms13081885 (PMC12388204; doi:10.3390/microorganisms13081885)
Supplement: Supplementary file 1 [file microorganisms-13-01885-s001.zip › microorganisms-3733573-supplementary.pdf]

FIGURE CAPTION

Figure S1. Comparison of the beta diversity of the two base growing media conditions by genus (A, C) and phylum (B, D). The Sorensen metric accounts for presence/absence (A, B), whereas the Bray-Curtis beta-diversity metric accounts for abundance as well as presence (C, D). This is the same data as in Figure 2 but analyzed focusing on overall growth media matrix.

Figure S2. Comparison of the beta diversity of the growth media conditions within the compost-based growth media by phylum (A, B) and genus (C, D). The Sorensen metric accounts for presence/absence (A, C), whereas the Bray-Curtis beta-diversity metric accounts for abundance as well as presence (B, D). This is the same data as in Figure 2 but analyzed without the other growth media (PL, PLI, PLV) samples included.

Figure S3. Comparison of the beta diversity of the growth media conditions within the Peat Lite-based growth media by phylum (A, B) and genus (C, D). The Sorensen metric accounts for presence/absence (A, C), whereas the Bray-Curtis beta-diversity metric accounts for abundance as well as presence (B, D). This is the same data as in Figure 2 but analyzed without the other growth media (C, CI, CV, CVW) samples included.

Figure S1

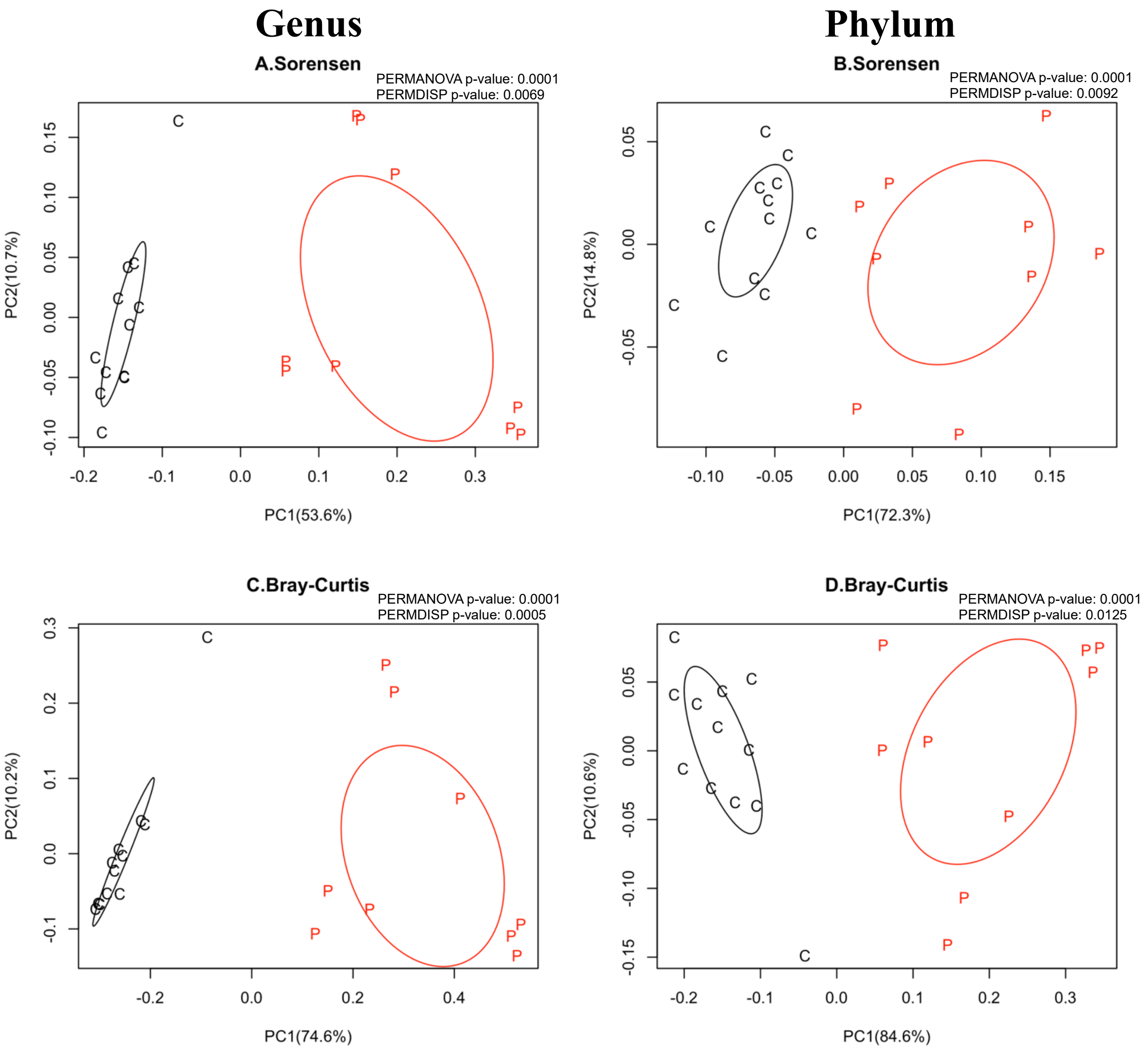
Genus
Phylum
A.Sorensen
PERMANOVA p-value: 0.0001
PERMDISP p-value: 0.0069
PC1(53.6%)
PC2(10.7%)
-0.2 -0.1 0.0 0.1 0.2 0.3
-0.10 -0.05 0.00 0.05 0.10 0.15
B.Sorensen
PERMANOVA p-value: 0.0001
PERMDISP p-value: 0.0092
PC1(72.3%)
PC2(14.8%)
-0.10 -0.05 0.00 0.05 0.10 0.15
-0.05 0.00 0.05
C.Bray-Curtis
PERMANOVA p-value: 0.0001
PERMDISP p-value: 0.0005
PC1(74.6%)
PC2(10.2%)
-0.2 0.0 0.2 0.4
-0.1 0.0 0.1 0.2 0.3
D.Bray-Curtis
PERMANOVA p-value: 0.0001
PERMDISP p-value: 0.0125
PC1(84.6%)
PC2(10.6%)
-0.2 -0.1 0.0 0.1 0.2 0.3
-0.15 -0.10 -0.05 0.00 0.05

Figure S2

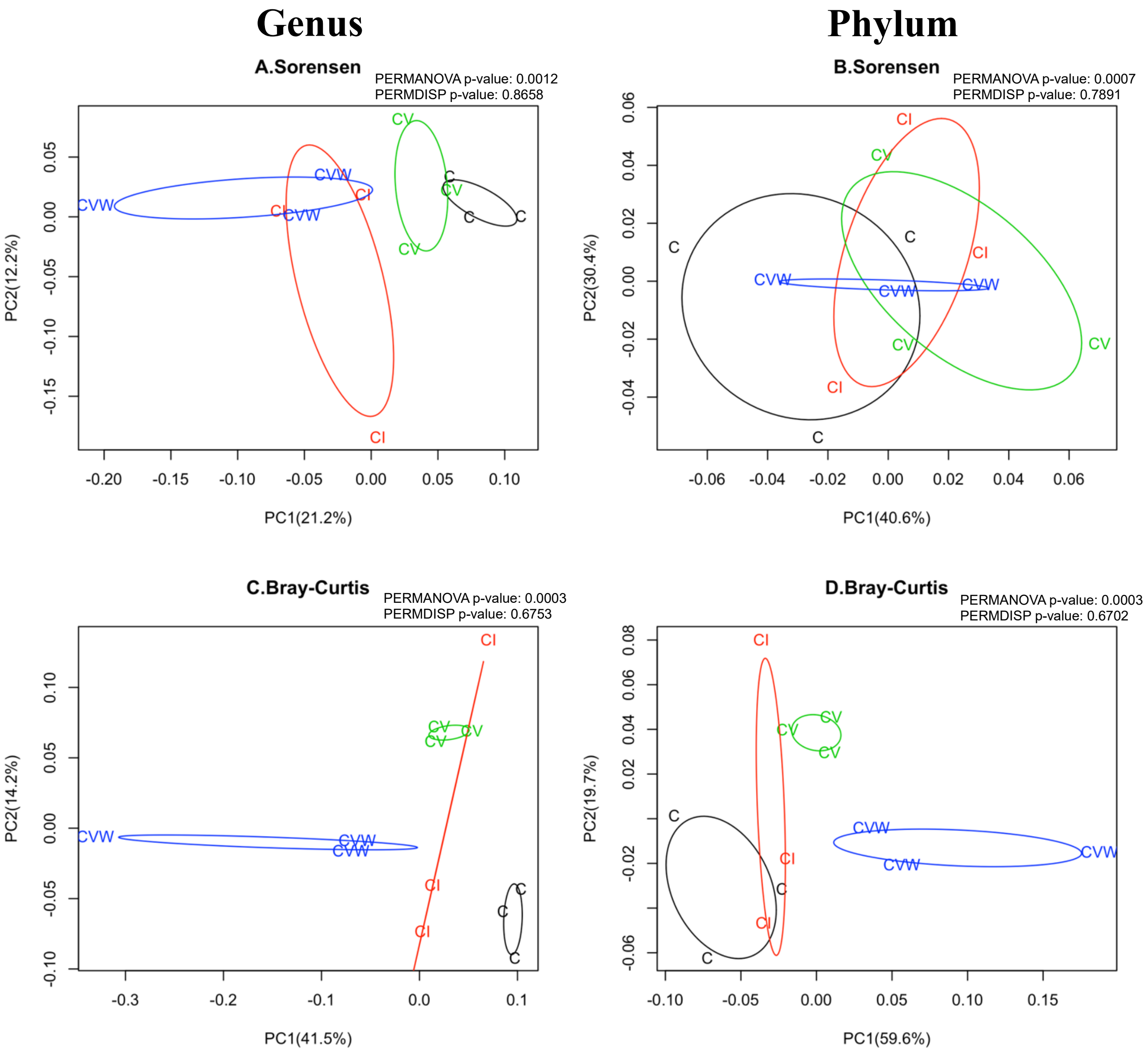
Genus
Phylum
A.Sorensen
PERMANOVA p-value: 0.0012
PERMDISP p-value: 0.8658
PC1(21.2%)
PC2(12.2%)
B.Sorensen
PERMANOVA p-value: 0.0007
PERMDISP p-value: 0.7891
PC1(40.6%)
PC2(30.4%)
C.Bray-Curtis
PERMANOVA p-value: 0.0003
PERMDISP p-value: 0.6753
PC1(41.5%)
PC2(14.2%)
D.Bray-Curtis
PERMANOVA p-value: 0.0003
PERMDISP p-value: 0.6702
PC1(59.6%)
PC2(19.7%)
C
CI
CV
CVW

Figure S3

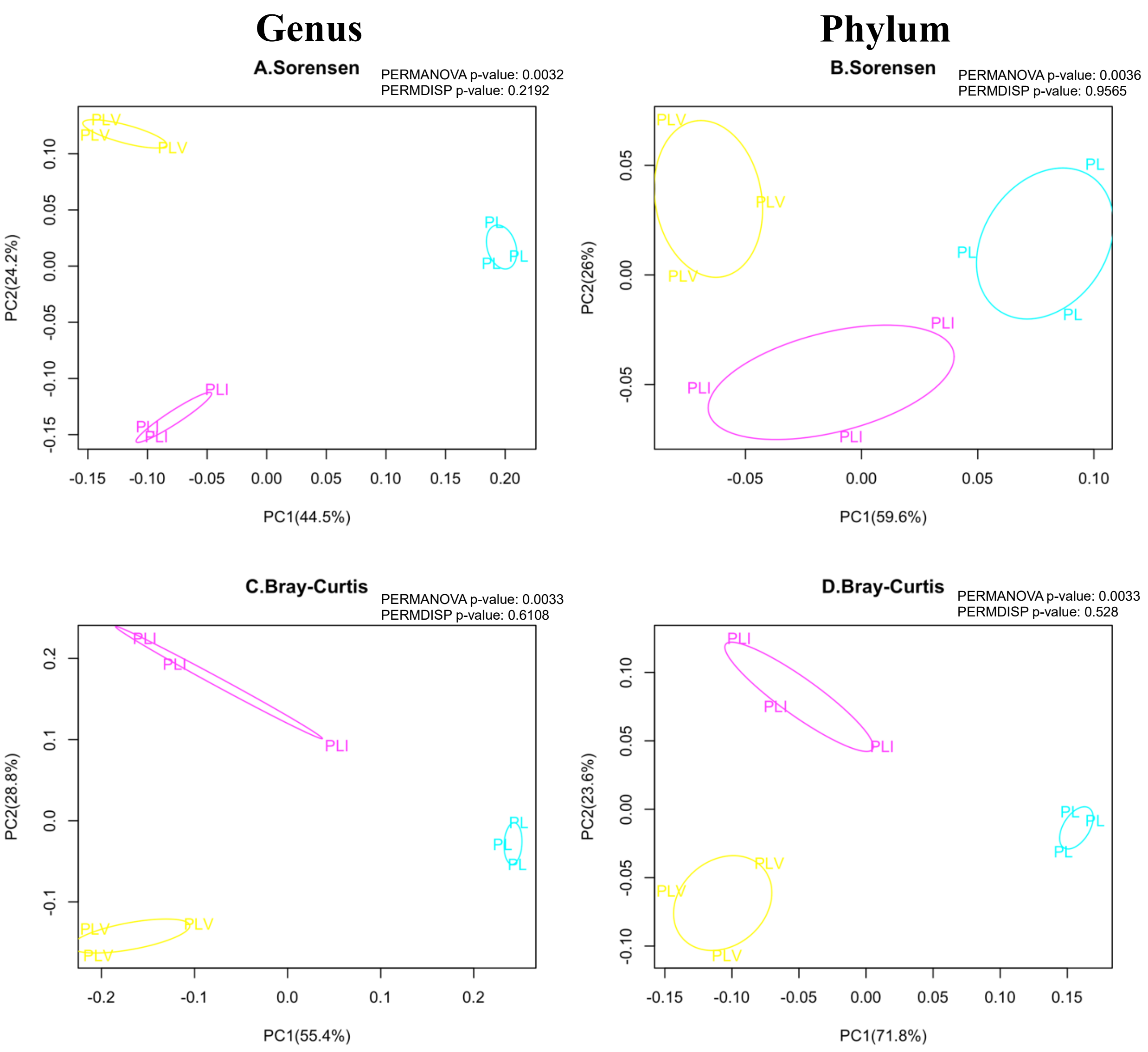
Genus
Phylum
A.Sorensen
PERMANOVA p-value: 0.0032
PERMDISP p-value: 0.2192
PC1(44.5%)
PC2(24.2%)
B.Sorensen
PERMANOVA p-value: 0.0036
PERMDISP p-value: 0.9565
PC1(59.6%)
PC2(26%)
C.Bray-Curtis
PERMANOVA p-value: 0.0033
PERMDISP p-value: 0.6108
PC1(55.4%)
PC2(28.8%)
D.Bray-Curtis
PERMANOVA p-value: 0.0033
PERMDISP p-value: 0.528
PC1(71.8%)
PC2(23.6%)
PLV
PLI
PL

Table S1. Individual taxa comparison in PL, PLI, and PLV at the phylum level.

Taxa name	Overall	PL	PLI	PLV	p-value
Proteobacteria	45.1 ± 10.9	58.9 ± 1.4^{a}	38.4 ± 5.8^{b}	37.9 ± 3^{b}	<0.0001
Planctomycetes	12.7 ± 4.2	8 ± 1.3^{a}	17.1 ± 1.7^{b}	12.8 ± 2.1^{c}	<0.0001
Acidobacteria	9.2 ± 5.6	3.8 ± 0.5^{a}	8 ± 1.8^{b}	15.8 ± 3.1^{c}	<0.0001
Chloroflexi	2.7 ± 2	0.3 ± 0.1^{a}	2.7 ± 0.2^{b}	5 ± 0.6^{c}	<0.0001
Bacteroidetes	13.2 ± 2.1	14.2 ± 1.4^{a}	14.4 ± 1.9^{a}	10.9 ± 0.6^{b}	<0.0001
Actinobacteria	4.6 ± 1.4	4.5 ± 0.5^{ab}	3.7 ± 0.7^{a}	5.7 ± 2^{b}	0.0200
Verrucomicrobia	6.4 ± 2.3	5.6 ± 0.5^{a}	9.2 ± 1.7^{b}	4.4 ± 0.7^{a}	<0.0001
Armatimonadetes	1.3 ± 0.7	1.6 ± 0.1^{ab}	1.6 ± 1^{a}	0.7 ± 0.1^{b}	0.0040

Values reported as mean ± SD

Values in a row that do not contain the same superscript are significantly different, p<0.05

P-values were Benjamini-Hochberg corrected.

Table S2. Individual taxa comparison in C, CI, CV, and CVW at the phylum level.

Taxa name	Overall	C	CI	CV	CVW	p-value
Proteobacteria	22.6 ± 2.3	21.4 ± 1.9^{a}	21.1 ± 1.5^{a}	25.3 ± 2.7^{b}	22.7 ± 0.9^{ab}	0.0050
Planctomycetes	15.2 ± 3.2	12.5 ± 0.8^{a}	14.4 ± 1.5^{a}	14.5 ± 2.4^{a}	19.4 ± 2.8^{b}	<0.0001
Acidobacteria	22.9 ± 5.1	28.5 ± 3.5^{a}	24.2 ± 4.8^{abc}	19.9 ± 0.9^{bc}	18.8 ± 4.8^{c}	0.0050
Chloroflexi	15.6 ± 3.2	15.3 ± 2.6^{a}	17.9 ± 4.4^{a}	16.7 ± 1.4^{a}	12.5 ± 2.3^{b}	0.0200
Bacteroidetes	7.3 ± 2.4	5.8 ± 2.1^{a}	6.5 ± 0.5^{a}	6.6 ± 0.9^{a}	10.5 ± 2.5^{b}	0.0004
Actinobacteria	2.9 ± 0.5	3 ± 0.8^{a}	3 ± 0.4^{a}	2.9 ± 0.3^{a}	2.7 ± 0.7^{a}	0.8000
Verrucomicrobia	3.8 ± 1.5	3 ± 0.3^{a}	3 ± 0.4^{a}	3.9 ± 0.5^{ab}	5.2 ± 2.6^{a}	0.0060
Firmicutes	2.2 ± 1	2.9 ± 1.3^{a}	2.4 ± 1.6^{a}	1.7 ± 0.3^{a}	2 ± 0.2^{a}	0.2000
Gemmatimonadetes	2.4 ± 0.6	2.7 ± 0.3^{a}	2.4 ± 0.4^{ab}	2.8 ± 0.7^{a}	1.7 ± 0.5^{b}	0.0050
Bacteria	1.7 ± 0.4	1.7 ± 0.2^{a}	1.8 ± 0.3^{ab}	2 ± 0.1^{a}	1.2 ± 0.2^{b}	<0.0001

Values reported as mean ± SD

Values in a row that do not contain the same superscript are significantly different, p<0.05

P-values were Benjamini-Hochberg corrected.

Table S3. Individual taxa comparison in PL, PLI, and PLV at the genus level.

Taxa name	Overall	PL	PLI	PLV	p-value
Subgroup 6 ge	5.9 ± 5.7	0.8 ± 0.2[a]	3.9 ± 1.3[b]	12.9 ± 2.9[c]	<0.0001
Uncultured Pirellulaceae	1.2 ± 0.3	1 ± 0.4[a]	1.6 ± 0.2[b]	1.1 ± 0.1[a]	0.0030
Flavobacterium	2.5 ± 1.1	2.4 ± 0.3[a]	3.6 ± 1.1[b]	1.4 ± 0.4[c]	<0.0001
Burkholderiaceae unclassified	1.1 ± 0.2	1.2 ± 0.1[a]	1.2 ± 0.1[a]	0.8 ± 0[b]	0.0006
SH PL14	2.2 ± 0.8	2.2 ± 0.9[ab]	3 ± 0.2[a]	1.5 ± 0.5[b]	0.0040
Uncultured Micropepsaceae	2.3 ± 0.9	3.4 ± 0.5[a]	1.7 ± 0.8[b]	1.8 ± 0.2[b]	<0.0001
Pedobacter	1.8 ± 0.6	2.6 ± 0.1[a]	1.3 ± 0.4[b]	1.5 ± 0[b]	<0.0001
Prosthecobacter	1.1 ± 0.3	1.3 ± 0.1[a]	1.2 ± 0.4[a]	0.9 ± 0.2[b]	0.0300
Streptomyces	1 ± 0.4	1.3 ± 0.4[a]	0.9 ± 0.5[a]	0.8 ± 0.1[a]	0.0900
Burkholderia Caballeronia Paraburkholderia	6.3 ± 2.8	9.6 ± 1.3[a]	4.9 ± 2.3[ab]	4.6 ± 1.2[b]	0.0006
Rhodanobacteraceae unclassified	2 ± 1.1	3.3 ± 0.4[a]	1.3 ± 0.5[b]	1.4 ± 0.6[b]	<0.0001
Rhodanobacter	6.3 ± 2.8	9.8 ± 1[a]	4.5 ± 1.6[b]	4.5 ± 0.3[b]	<0.0001
Mucilaginibacter	1.5 ± 0.8	2.4 ± 0.2[a]	1 ± 0.5[b]	1 ± 0.3[b]	<0.0001
Caulobacter	1 ± 0.6	1.7 ± 0.2[a]	0.7 ± 0.3[b]	0.6 ± 0.1[b]	<0.0001
Sphingopyxis	1.1 ± 0.4	1.5 ± 0.2[a]	0.9 ± 0.4[b]	0.9 ± 0.3[b]	0.0100
Ramlibacter	1.3 ± 0.5	2 ± 0.2[a]	1.1 ± 0.2[b]	0.9 ± 0.1[b]	<0.0001

Values reported as mean ± SD

Values in a row that do not contain the same superscript are significantly different, p<0.05

P-values were Benjamini-Hochberg corrected.

Table S4. Individual taxa comparison in C, CI, CV, and CVW at genus level.

Taxa name	Overall	C	CI	CV	CVW	p-value
A4b ge	3.2 ± 0.9	2.9 ± 0.2[ab]	2.9 ± 1[ab]	4.2 ± 1[a]	2.9 ± 0.4[b]	0.0300
Bacteria unclassified	1.7 ± 0.4	1.7 ± 0.2[a]	1.8 ± 0.3[a]	2 ± 0.1[a]	1.2 ± 0.1[b]	<0.0001
Chryseolinea	1.6 ± 0.4	1.4 ± 0.4[a]	1.6 ± 0.4[a]	1.8 ± 0.5[a]	1.6 ± 0.4[a]	0.6000
Gammaproteobacteria unclassified	1.1 ± 0.3	1.1 ± 0.1[ab]	1.1 ± 0.3[ab]	1.2 ± 0.4[a]	0.8 ± 0.1[b]	0.1000
Pedosphaeraceae ge	2.1 ± 0.5	1.9 ± 0.2[ac]	1.9 ± 0.2[ac]	2.8 ± 0.5[b]	1.7 ± 0.5[c]	0.0004
Pir4_lineage	1.8 ± 0.5	1.7 ± 0.1[a]	1.3 ± 0.3[a]	1.7 ± 0.2[a]	2.6 ± 0.3[b]	<0.0001
Pirellula	1.2 ± 0.2	1.1 ± 0.1[a]	1.3 ± 0.2[a]	1.2 ± 0.2[a]	1.3 ± 0.2[a]	0.5000
SBR1031 ge	1.5 ± 0.8	1.4 ± 0.2[ab]	2.1 ± 1.4[a]	1.5 ± 0.2[ab]	1 ± 0.7[b]	0.1000
SH PL14	1.6 ± 0.5	1.1 ± 0.1[a]	1.5 ± 0.4[abc]	1.6 ± 0.3[bc]	2.1 ± 0.4[c]	0.0004
Subgroup 6 ge	18.6 ± 4.8	23.6 ± 3[a]	20 ± 4[ab]	15.7 ± 1.3[bc]	15 ± 5[c]	0.0100
Uncultured Anaerolineaceae	3.7 ± 1.4	3.4 ± 0.8[a]	4.5 ± 2.2[a]	4 ± 0.3[a]	2.7 ± 1.4[a]	0.2000
Uncultured Gemmatimonadaceae	1 ± 0.2	1.2 ± 0.1[a]	1 ± 0.1[ab]	1.1 ± 0.2[a]	0.8 ± 0.1[b]	0.0100
Uncultured Microscillaceae	1.2 ± 0.3	0.9 ± 0.1[a]	1.2 ± 0.2[ab]	1.3 ± 0.2[ab]	1.5 ± 0.5[b]	0.0200
Uncultured Pirellulaceae	1.4 ± 0.4	1.3 ± 0.2[a]	1.4 ± 0.3[a]	1.2 ± 0.4[a]	1.9 ± 0.3[b]	0.0010
WD2101 soil group ge	2 ± 0.5	1.9 ± 0.5[a]	1.9 ± 0.6[a]	2.4 ± 0.5[a]	2 ± 0.3[a]	0.4000
X11 24 ge	1 ± 0.3	1.4 ± 0.1[a]	0.8 ± 0[bc]	1 ± 0[abc]	0.9 ± 0.4[c]	0.0004

Values reported as mean ± SD

Values in a row that do not contain the same superscript are significantly different, $p<0.05$

P-values were Benjamini-Hochberg corrected.
